# Recent advances in the anti-tumor activities of saponins through cholesterol regulation

**DOI:** 10.3389/fphar.2024.1469392

**Published:** 2025-01-07

**Authors:** Min Jiang, Chao Hong, Wenkui Zou, Zheng Ye, Lu Lu, Yun Liu, Tong Zhang, Yue Ding

**Affiliations:** ^1^ School of Pharmacy, Shanghai University of Traditional Chinese Medicine, Shanghai, China; ^2^ State Key Laboratory of Integration and Innovation of Classic Formula and Modern Chinese Medicine, Shanghai University of Traditional Chinese Medicine, Shanghai, China; ^3^ National Innovation Platform for Medical Industry-Education Integration, Shanghai University of Traditional Chinese Medicine, Shanghai, China

**Keywords:** saponins, anti-tumor, cell membrane, cholesterol, metabolism

## Abstract

Abnormal cholesterol metabolism has become a popular therapeutic target in cancer therapy. In recent years there has been a surge in interest in the anti-tumor activities of saponins, particularly their ability to disrupt cholesterol homeostasis in tumor cells. Cholesterol regulation by saponins is a complex process that involves multiple mechanisms. However, there are now a notable dearth of comprehensive reviews addressing their anti-tumor effects through cholesterol modulation. This review will explore the intricate mechanisms by which saponins regulate cholesterol, including modulation of synthesis, metabolism, and uptake, as well as complex formation with cholesterol. It will also outline how saponins exert their anti-cancer activities through cholesterol regulation, enhancing cytotoxicity, inhibiting tumor cell metastasis, reversing drug resistance, inducing immunotoxin macromolecule escape, and ferroptosis. This comprehensive analysis offers insights into the potential for the use of saponins anti-tumor therapies and their combinations with other drugs, advancing the understanding of their effects on cancer cells.

## 1 Introduction

Abnormal cholesterol metabolism has become a prominent characteristic of cancer (Xu et al., 2020). Numerous preclinical and clinical studies, along with data from The Cancer Genome Atlas (TCGA), reveal dysregulation of genes and proteins associated with cholesterol metabolism in various human cancer cells (Huang et al., 2020; Schade et al., 2020). This highlights the significance of targeting cholesterol metabolism as a crucial therapeutic intervention for cancer treatment. Cholesterol not only triggers oncogenic signaling in cancer cells but also promotes tumorigenesis through its precursors and derivatives (Ediriweera, 2022). Recent evidence suggests that the metabolism of cholesterol plays an important role in the tumor microenvironment (Jin et al., 2023). Consequently, considerable efforts have been dedicated to identifying potential methods to break cholesterol homeostasis, with the aim of developing effective anti-cancer strategies.

Statin drugs, approved by the FDA for cholesterol reduction, have been successfully utilized for cancer treatment (Jiang et al., 2021). HMGCR, a main therapeutic target of statins, has been explored as a potential anti-cancer target. Also, combination therapy involving statins and other chemotherapeutic agents has shown promising clinical outcomes compared to statin monotherapy (Rao et al., 2023; Sahebkar et al., 2021). Although clinical investigations have demonstrated favorable anti-cancer effects of lower statin doses compared to clinically used doses, resistance to statins has still been observed in certain cancer cell types. Remarkably, some tumor cell lines such as MCF-7 and T47D have shown drug resistance to statins due to a feedback response triggered by HMGCR activation (Jawaid et al., 2010; Gobel et al., 2019). This indicates that the anti-tumor effects of treatment with statins may not usually be encouraging in all cases. Further, it is important to note that statins do not exhibit cholesterol-lowering effects in mice (Huang et al., 2023), making it more difficult to carry out preclinical research.

Traditional Chinese Medicine (TCM) has been extensively used in East Asia for cancer treatment, either as a standalone approach or as a complementary therapy. TCM has shown clear advantages over conventional therapies in terms of minimal side effects, reduced toxicity, and lower economic burden (Zhang et al., 2021). In recent years, numerous studies have provided evidence that certain Chinese herbs could potentially break the cellular cholesterol homeostasis, making them valuable for treating cancers (Zheng et al., 2023; Cao et al., 2023). With the progress of contemporary science and technology, researchers have successfully isolated and extracted the active ingredients from these Chinese herbs. Further studies have revealed that the cholesterol-regulating mechanisms of these active ingredients differ from those of classical Western medications. Consequently, the combination of these with medicine offers a novel approach for clinically addressing cancers.

**FIGURE 1:**
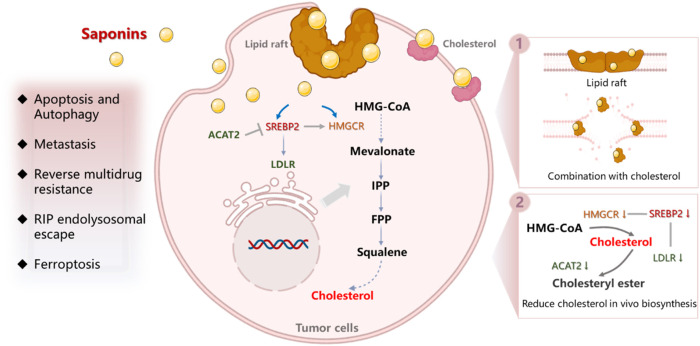
The mechanism of saponins disturbance of cholesterol balance in cancer cells.

Saponins, being representative active ingredients in TCM, exhibit a wide range of pharmacological activities (Man et al., 2010), including cardiovascular protective effects (Wang et al., 2020), anti-inflammatory effects (Passos et al., 2022), anti-viral activity (Sharma et al., 2021), and immunoregulatory effects (Yang et al., 2017). More recently, many important studies have reported that saponins exhibit significant anti-cancer activity, such as inhibition of cell proliferation, suppression of metastasis and angiogenesis (Xu et al., 2016), as well as reversal of multi-drug resistance (MDR) effects (Qu et al., 2022; Eid et al., 2012). Furthermore, they have been found to mitigate the adverse effects associated with radiotherapy and chemotherapy (Zhang et al., 2021; Sun et al., 2022), indicating the promising potential of saponins in anti-cancer therapy. Of particular interest is their ability to act against a tumor by disturbing cholesterol balance in tumor cells. They may not only have an influence on cholesterol synthesis, metabolism (Malinow, 1984), and absorption in tumor tissue (Xie et al., 2024) but also affect the cholesterol content, distribution and function in the cell membrane (Bottger and Melzig, 2013), finally inducing cell death directly or enhancing the anti-tumor effect of other drugs. Compared to current clinical cholesterol-disturbing drugs such as statins, saponins possess the advantage of modulating cholesterol through diverse pathways and mechanisms, consequently restricting tumor growth with less likelihood of causing rug resistance. However, a critical gap exists in the literature, as comprehensive and systematic review articles summarizing the anti-tumor or synergistic anti-tumor effects of saponins via cholesterol regulation are lacking. This paucity of information potentially hinders the widespread use of saponins in the field of anti-tumor and in combination with other drugs. Therefore, we have conducted a review to provide more detailed information on the influence and mechanisms of saponins on cholesterol reprogramming, which will hopefully contribute to a better understanding of saponins’ effects on cancer cells as shown in Figure 1.

## 2 Saponins regulate the expression of key enzymes or proteins related to cholesterol synthesis and metabolism

Cholesterol, its precursors, and metabolites play crucial roles in the development, progression, and invasion of tumors. Consequently, the regulation of cholesterol metabolism in tumor cells has emerged as a promising anti-tumor therapeutic strategy (Liu et al., 2023). Key factors, such as metabolic enzymes and transporters, in the cholesterol synthesis and metabolism pathway of tumor cells have the potential to serve as regulatory targets (Cheng et al., 2018). Currently, the arsenal of clinically used drugs to target cholesterol regulation includes statins (Jiang et al., 2021), which inhibit HMG-CoA reductase (HMGCR), and Ezetimibe (Lamb, 2020), which selectively inhibits the intestinal absorption transporter NPC1L1. In addition to these, numerous drug candidates are being investigated in preclinical studies for their potential to modulate cholesterol metabolism. These include SREBP inhibitors (Guo et al., 2014) such as PF-429242 (Raini et al., 2021) and 25-hydroxycholesterol (Dang et al., 2017), as well as Dendrogenin A targeting cholesterol epoxide hydrolase (ChEH) (Dalenc et al., 2015). However, with significant side effects and clinical toxicity issues, this targeting treatment strategy is still in its early stages.

**FIGURE 2:**
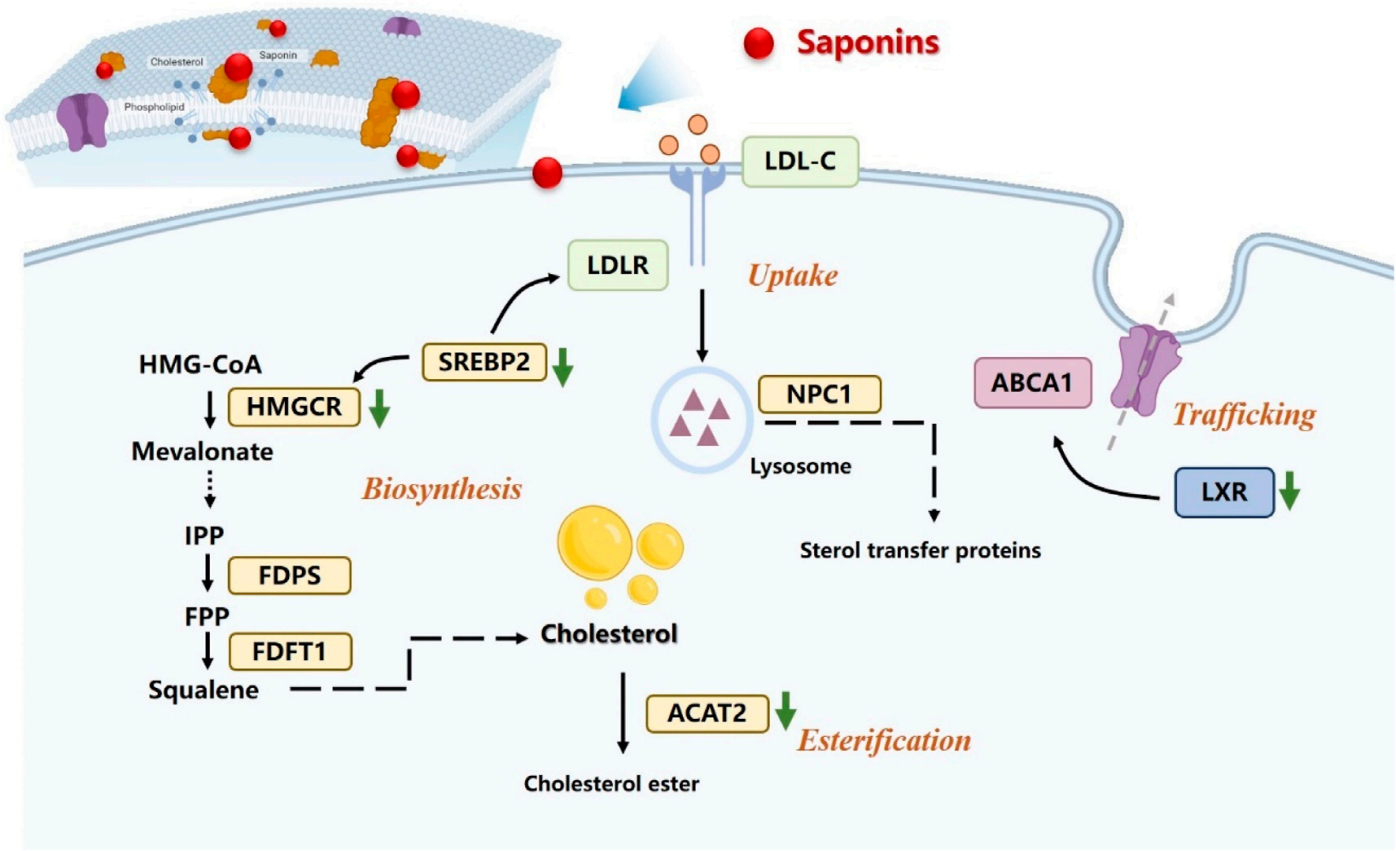
Saponins regulation of the intracellular cholesterol metabolism pathway.

Saponins, specifically, have been shown to regulate various aspects of lipid metabolism, including the synthesis, absorption, transport, and metabolism of cholesterol, as shown in Figure 2. These saponins primarily target key regulators involved in lipid metabolism, such as HMGCR, lipoprotein lipase (LPL), cholesterol ester transfer protein (CEPT), cholesterol 7α-hydroxylase (CYP7A1), peroxisome proliferator-activated receptors (PPARs), sterol regulatory element-binding protein (SREBP), and liver X receptor α (LXRα), among others. Saponins therefore hold great promise as potential anti-tumor therapeutics that exert their effects through the intricate pathways of cholesterol metabolism.

### 2.1 Cholesterol biosynthesis

#### 2.1.1 HMG-CoA reductase (HMGCR)

HMGCR is able to control the rate-limiting step in cholesterol production, making it a crucial part of the pathway (Sharpe and Brown, 2013). Abnormal activation of HMGCR has been observed in various tumors, leading to excessive tumor proliferation and metabolic reprogramming. Raju and Bird (2007) discovered that diosgenin, an active saponin isolated from *Trigonella foenum-graecum L*., can suppress the expression of HMGCR and induce apoptosis in HCT-116 human colon carcinoma cells. Their observation identified that cholesterol homeostasis was closely involved in the growth suppressive or apoptotic activity of diosgenin. However, the depictions of the role of saponins in regulating HMGCR in the studies are relatively general.

#### 2.1.2 Sterol regulatory element-binding protein 2 (SREBP2)

SREBPs are key regulators of lipid synthesis and their elevated expression in various cancer types further supports tumor proliferation and metastasis. Therefore, targeting SREBPs with saponins, which possess cholesterol-regulating properties, has been proposed as a potential approach for exerting anti-cancer effects. In a separate study, it was reported that methyl protodioscin (MPD) could inhibit the transcription of SREBP1c and SREBP2, leading to decreased expression of HMGCR, acetyl CoA carboxylase (ACC) and fatty acid synthase (FAS) genes involved in cholesterol and fatty acid synthesis in HepG2 cells. The rationale for the mechanism involved the suppression of SREBP1c and SREBP2 transcription through the reduction of microRNA 33a/b levels. PPD also exhibits a similar effect in THP-1 macrophages by reducing HMGCR, FAS, and ACC mRNA levels and increasing low-density lipoprotein receptor expression through the suppression of PCSK9 levels. Furthermore, Chavez-Santoscoy et al. (2014) reported that soyasaponin Af, the primary saponin found in Black Bean, significantly reduces the expression levels of HMGCR, FAS and SREBP1c in the liver, and stimulated the expression of reverse cholesterol transporters CYP7A1 and ABCG5/ABCG8. Moreover, it has been reported that Timosaponin AⅢ (Tim-AIII) can induce autophagy in liver and breast cancer through the mTOR signaling pathway (King et al., 2009). By inhibiting autophagy and maintaining endoplasmic circulation, mTORC1 stops autophagosomes and endosomes from arriving lysosomes, thereby reducing ER cholesterol concentrations, and activating the expression of SREBP-2 (Peterson et al., 2011).

#### 2.1.3 Farnesyl-diphosphate farnesyltransferase 1 (FDFT1)

FDFT1 is a crucial factor that determines the direction of sterol synthesis and is located in the endoplasmic reticulum, reactions occur downstream of the HMGCR production pathway from FPP(Tuzmen et al., 2019). FDFT1 acts as a key regulator of the mevalonate pathway, directing intermediates to the sterol branch rather than the nonsterol branch, which results in a specialized cholesterol biosynthetic step (Nakae et al., 2021). Lu et al. (2020) found that when miR-4425 mimics or FDFT1 siRNA were transfected into 20(S)-Rg3-treated ovarian cancer cells they could counteract the anti-tumor activity of the ginsenoside Rg3. Their study showed that Rg3 exerted anti-ovarian cancer activity by down-regulating the expression of oncogene miR-4425, thereby affecting the expression of FDFT1. Although accelerated cholesterol metabolism is associated with cancer development and progression, it is inconclusive whether FDFT1 is a tumor-suppressor gene or a candidate oncogene (Weng M. L. et al., 2020). Among them, the verification of the direct effect of saponins on FDFT1 is not yet complete, and is only based on phenotypic data. However, it holds certain significance for in-depth research in the future.

### 2.2 Cholesterol esterification

#### 2.2.1 Acetyl coenzyme an acetyltransferase 2 (ACAT2)

Studies have also shown that saponins can play an anti-tumor role by regulating key target factors in cholesterol storage pathways. Acyl CoA: cholesterol acyltransferase (ACAT) is an intracellular enzyme that relies upon cholesterol and long-chain fatty acyl CoA as substrates for producing cholesteryl esters (Kwon et al., 1999). Typically, ACAT2 was a critical molecule in the cholesterol absorption progress. It is widely accepted that ingesting large amounts of dietary cholesterol can lead to hypercholesterolemia and atherosclerosis, which can induce cancer production, thereby the inhibition of ACAT is an attractive therapeutic target (Weng M. et al., 2020). Kwon et al. (1999) study showed that ginsenosides could inhibit ACAT *in-vitro* mildly, and significantly that the sapogenins showed strong inhibitory activity on microsomal ACAT, with an inhibition rate of 72%–80%. In our previous research (Jiang et al., 2022),we found that Polyphyllin I also had a certain inhibitory effect on ACAT2 in HepG2 cells, which could effectively inhibit the esterification of cholesterol and thus inhibit the excessive storage of the molecule.

### 2.3 Cholesterol uptake

#### 2.3.1 Low-density lipoprotein receptor (LDLR)

Cholesterol is an important part of cell membrane, an important component of maintaining the integrity and fluidity of cell membrane, and an important substance for regulating membrane fluidity, cell adhesion to extracellular matrix and signal transduction initiation, helping cells to function and maintain body health (Xu et al., 2020). The liver is responsible for producing most of the body’s cholesterol, which is then transported to other organs through low-density lipoprotein (LDL) particles that package cholesterol (Go and Mani, 2012). The expression of LDLR is tightly regulated in response to intracellular cholesterol levels via transcriptional and post-translational pathways, with SREBP-2 controlling the transcription of the LDLR gene (Kartawijaya et al., 2016). Choi et al. (2020) aimed to elucidate the molecular mechanisms underlying Platycodin D (PD), a triterpenoid saponin extracted from Platycodin (PG), on LDLR expression and LDL-C uptake in hepatocytes, and to elucidate the molecular mechanism of its cholesterol-lowering effect. They discovered that PD upregulated cell surface LDLR expression in HepG2 cells, leading to increased uptake of LDL-C particles. This finding suggests that PD induces both LDLR expression and uptake by inhibiting the LXR-IDOL pathway. Additionally, Lee et al. (2022) found that compared with normal astrocytes, PD enhanced the expression of LDLR on the cell surface and accelerated the uptake of exogenous LDL-C, especially in GBM cells. In addition, PD-induced LDLR increases cholesterol uptake and subsequent lysosomal cholesterol accumulation lead to autophagy inhibition and GBM cell death. Therefore, the anti-cancer effect of PD is related to the abundance of LDLRs in GBM cells.

### 2.4 Cholesterol transport

#### 2.4.1 ABC subfamily A member 1 (ABCA1)

The trafficking of specific molecules across cell membranes is a critical function of all living organisms. The processes are always mediated by associated transporters (Liu, 2019). The ATP-binding box (ABC) transporters on the cell membrane are mainly involved in the cholesterol efflux, which is mainly composed of ABC subfamily A member 1 (ABCA1) and ABC subfamily G members 1, 5 and 8 (ABCG1, ABCG5, ABCG8) (Nobili et al., 2020) and ABCA1 is also closely related to the treatment of tumors. Ma et al. (2015) found that in THP-1 macrophages, methyl protodioscin (MPD) suppressed the transcription of SREBP1c and SREBP2, and decreased levels of microRNA 33a/b hosted in the introns of SREBPs, leading to a reciprocal increase ABCA1 levels and cholesterol efflux. Additionally, Gai et al. (2019) demonstrated that pseudoprotodioscin (PPD) increases ABCA1 protein and mRNA levels in HepG2 cells, promoting ApoA-1-mediated cholesterol efflux.

#### 2.4.2 Liver X receptor (LXR)

LXR plays an essential role in maintaining intracellular cholesterol homeostasis and belongs to the nuclear receptor family (Wang and Tontonoz, 2018). LXR forms heterodimers with retinoid X receptor (RXR) after activation by LXR agonists. This LXR-RER heterodimer binds to a coactivator, binds to LXR-responsive elements (LXREs) in the nucleus, and mediates the expression of genes associated with cholesterol metabolism, such as ABCA1, ABCG1, and inducible degraders of LDLR (IDOL) (Zelcer et al., 2009). Activation of LXR can inhibit the occurrence and development of cancers (Singh and Mehla, 2023). Qiu et al. (2022) showed that Ginsenosides saponins (Rb1, Rg1, Rg3, and CK) decreased intracellular cholesterol content and promoted cholesterol efflux in drug-resistant cells, leading to lipid rafts accumulated in specific regions of the cell membrane, finally increasing the cytotoxicity of temozolomide. They further concluded that the above results of cholesterol efflux and lipid raft redistribution were mainly due to these ginsenosides saponins being induced by stimulating LXRα. Wei et al. (2016) identified that Saikosaponin A could disrupt the formation of lipid rafts by depleting cholesterol and inhibiting TLR4 translocation into lipid rafts and thereby further activate the expression of LXRα, ABCA1 and ABCG1.

## 3 Saponins combine with cholesterol in membranes

**FIGURE 3:**
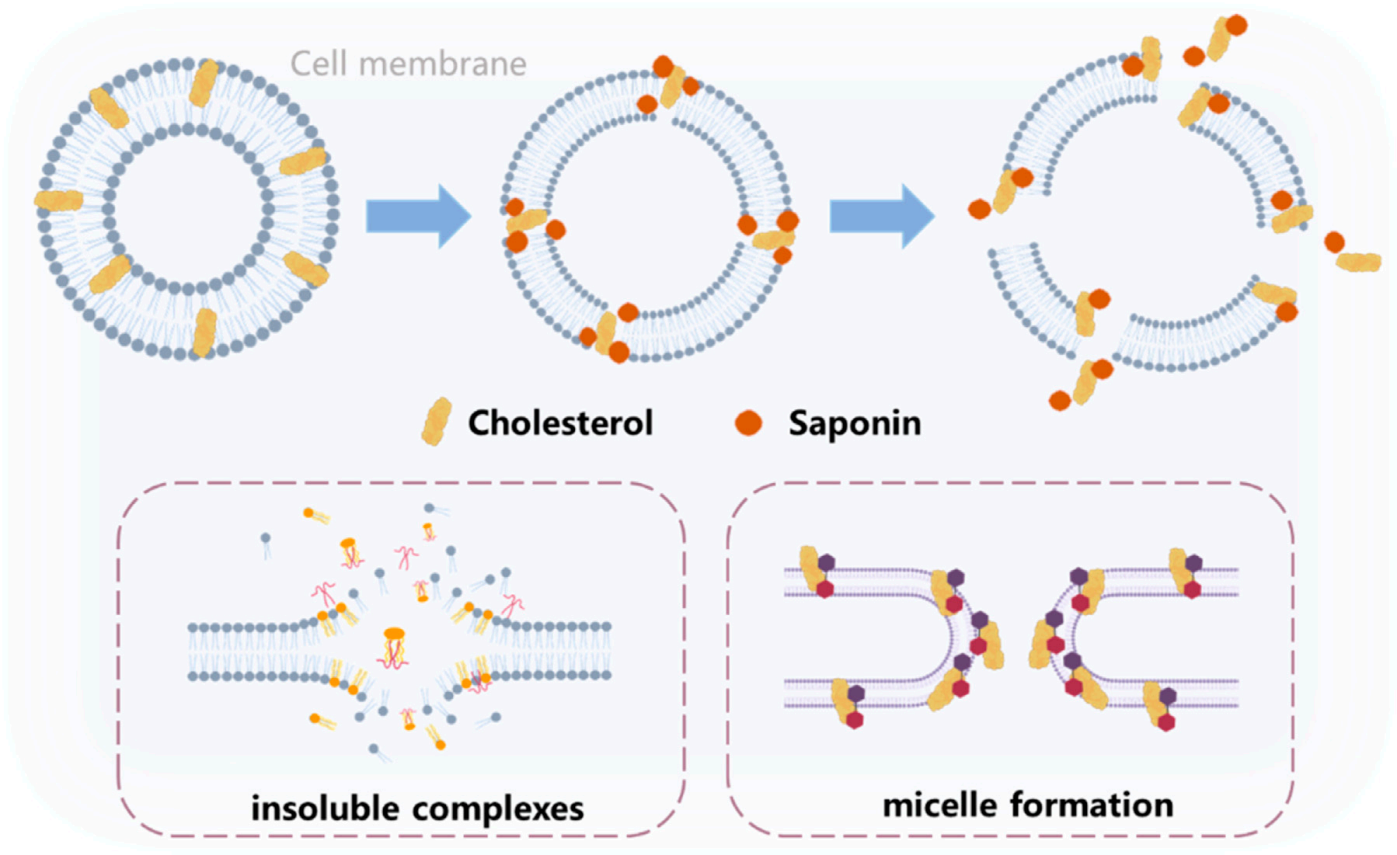
The interaction mechanism of cholesterol and saponins in the cell membrane.

Cell membrane cholesterol accounts for 90% of total cellular cholesterol. Saponins can not only regulate the metabolism of cholesterol in tumor cells, but also exert an anti-tumor effect by directly binding membrane cholesterol. Due to their amphiphilic structure, many saponins have strong surface activity, which can allow them to bind to cell membrane cholesterol in various ways to exert anti-tumor effects on cell membrane permeability or allow other (often polar) molecules to reach the cytoplasm or nuclei (Fuchs et al., 2009). The specific methods are described below, and the mode of functioning are shown in Figure 3.

### 3.1 Saponins form insoluble complexes with cholesterol

In general, triterpenoid saponins and steroidal saponins could form stable complexes with cholesterol in the membrane. Studies have shown that saponins tend to bind to cholesterol on the erythrocyte membrane, leading to the formation of insoluble complexes. This alteration in osmotic pressure ultimately causes the distension and rupture of erythrocytes, resulting in hemolysis (de Groot and Muller-Goymann, 2016). Specifically, digitonin molecules have been demonstrated to bind to cholesterol in the membrane (Sudji et al., 2015). This binding process removes cholesterol from the membrane core and leads to the formation of cholesterol-digitonin complexes on the membrane surface (Frenkel et al., 2014). Additionally, α-tomatine could form similar complexes, while the aglycone tomatidine lacks this capability (Sucha et al., 2013). A study conducted by Gestetner et al. (1971) revealed that lucerne saponins have a high affinity for various sterols, particularly cholesterol and 7-dehydrocholesterol, forming water-insoluble complexes. In our research (Jiang et al., 2022), we observed that polyphyllin I can interact with cholesterol, resulting in the formation of an insoluble precipitate when dissolved in ethanol. Similarly, a separate study by Story et al. (1984) investigated the interactions between alfalfa saponins and cholesterol *in-vitro*. They observed that alfalfa saponins effectively bound cholesterol from both ethanol solutions and micellar suspensions. OSW-1 demonstrated cholesterol-dependent membrane activity comparable to digitonin (Malabed et al., 2017). The unique structure of OSW-1, with a partially acylated sugar chain attached at the D ring of the steroidal aglycone and a distinct flat triangular shape in its three-dimensional structure, contributes to its potent activity. This amphiphilic polarity likely differentiates the binding mode of OSW-1 to cholesterol from that of typical saponins like digitonin, which has a 3-O-sugar chain (Malabed et al., 2020).

### 3.2 The activity of saponins on micelle formation

Structurally, saponins consist of one or more hydrophilic sugar moieties and a lipophilic steroid or triterpenic component, making them amphiphilic compounds (Bottger et al., 2012). When saponins reach their critical micelle concentration (CMC), they aggregate in solution, coexisting with free monomers below the CMC. These aggregates are characterized by their “soft” or fluid-like nature, resulting from weak intermolecular forces such as hydrogen bonding, van der Waals, hydrophobic, or screened electrostatic interactions. When saponin aggregates encountered a compound capable of forming a more stable complex, they undergo dissociation and form a new complex with the compound.

Quillaja saponins can form mixed micelles with cholesterol, due to a 1000-fold enhancement in the solubility of the sterol (Orczyk and Wojciechowski, 2015). These mixed micelles are larger in size compared to pure saponin micelles, measuring 10 nm instead of 7 nm. They also exhibit a higher CMC concentration and aggregation number. It is important to note that cholesterol is a component of the lipid compartment within these micelles. In a study conducted by Demana et al. (2005) observed that the worm-like micelles assembled when different proportions of saponins and cholesterol were mixed. Additionally, *Saponaria officinalis L*. saponins were found to be capable of forming micelles with various shapes, including rod-like, worm-like, and spherical, in the presence of cholesterol (Lendemans et al., 2005).

### 3.3 The mechanisms and interactions of saponin with artificial membrane models containing cholesterol

Membranes provide an amphiphilic environment characterized through a hydrophobic gradient, which could provide a hydrophobic core. The artificial membrane models that have been used to study saponins has provided valuable insights into the interactions between these molecules and various membrane components within this amphiphilic environment. These studies have also offered valuable insights into the mechanisms underlying saponins’ interaction with cholesterol in these membrane models (Lorent J. H. et al., 2014).

Some saponins could insert themselves into the monolayers without cholesterol in many different conditions. However, in the case of α-tomatine (Story et al., 1984), it must be accompanied by cholesterol for it to be able to insert in the membrane layers. This insertion is effective only when the sterol’s hydroxyl function at the third position in the monolayer is in the β configuration (Bottger and Melzig, 2013). In another case, comprising cholesterol and egg yolk phosphatidylcholine, permanent insertion of digitonin was observed upon formation of an equimolar complex. In this study, crucial conditions for the combination effect of digitonin and cell membrane cholesterol was proposed. Firstly, with the increase of the ratios of the digitonin and cholesterol, both formed aggregated within the cell layer (Frenkel et al., 2014). Then rising to a certain molar ratio, an intermediate complex emerged, consisting of a mixture of aggregates and equimolecular compounds (Sudji et al., 2015). Lastly, after reaching a certain proportion, an equimolecular complex was produced in the membrane.

### 3.4 Permeabilizing activity

Numerous studies have consistently demonstrated the significant role of cholesterol in the process of membrane permeabilization induced by saponins. In the case of most saponins, cholesterol has been found to enhance or be essential for permeabilization. The permeabilization mechanisms of saponins can be summarized as follows: The first mechanism suggested that saponins interacted with sterols, forming equimolecular complexes within the membrane (Lorent J. et al., 2014). As the density of these complexes increased, a new lipid phase was generated due to the hydrophilic interactions with the saponins’ sugar moieties. The three-dimensional structure of these compounds could form some aggregate such as spherical buds or tubules. This rearrangement of the membrane structure ultimately led to membrane disruption. The second mechanism proposed a model for the formation of annular pores in a specific lipid composition consisting of POPC/DOPE/cholesterol, using avenacin A1 as the saponin (Armah et al., 1999). In this mechanism, the hydrophilic interaction between the saponins’ sugar moieties caused the aggregation of saponins and cholesterol. This aggregation subsequently led to the formation of pores in the bilayers, adopting a toroidal shape. The third mechanism involves α-hederin, another type of saponin, and describes a concentration-dependent permeabilization process. Under the concentrations of the CMC, the α-hederin saponins could bind to the outer-layer of the membrane (Lorent J. H. et al., 2014). This binding creates an area discrepancy and curvature between the inner and outer monolayers, leading to the formation of vesicles. As the concentration of α-hederin increases, the saponin, cholesterol, and phospholipids aggregate, forming wormlike structures within the membrane. These aggregates cause transient defects in the membrane and gradual permeabilization. The dimension of the sugar chain at the C3 position of the triterpenoid ring influences the formation of domains and the speed of permeabilization. The saponin of α-hederin could form pores in the membrane directly, resulting in the loss of structure of the layer material with concentrations exceeding the CMC. This suggests that micelles or aggregates could be formed by transferring large amounts of saponins near the membrane, which can directly interact with the membrane architecture.

The combination of saponins with cholesterol-enriched domains. Immediately results in the change in membrane permeabilization and the formation of increasingly larger cores (Korchowiec et al., 2015). Specifically, α-hederin showed a higher tendency to accumulate at the pore’s rim, stabilizing it through a reduction in line tension, by virtue of its amphiphilic nature. According to this model, the external monolayer experiences a positive curvature strain. The presence of two hydrophilic sugars in the saponin molecule gives it an axe-like shape, resulting in positive curvature stress in the trans bilayer direction. This stress leads to the formation of worm-like aggregates or macroscopic pores. The model considers the concentration dependent self-aggregation of saponins, their three-dimensional shape, affinity for cholesterol, and amphiphilicity.

The foregoing exposition elaborates in detail on the manner in which saponins interact and bind with cell membrane cholesterol. Nevertheless, the preponderance of extant literature primarily centers on the validation of these effects within *in vitro* experiments and proffers scant delineation of the specific merits, demerits, and applications of saponins. Whether this can furnish definite guidance for the anti-tumor application of saponins remains a subject that demands further exploration.

## 4 Anti-cancer activities of saponins based on cholesterol monitoring

The acquisition of oncogenes and the deletion of cancer suppressor genes are crucial aspects of reprogramming cholesterol metabolic pathways in cancer cells. In recent years, mounting evidence has emerged indicating that cholesterols are signaling molecules that promote tumor development (Ding et al., 2019). Given the pivotal roles played by cholesterol in cancer, there is currently a burgeoning interest in screening novel molecules and strategies targeting cholesterol within the field of cancer research, with Chinese pharmaceutical saponins offering new avenues for exploration.

Saponins have been widely considered because of their biological and pharmacological activities such as antibacterial, anti-inflammatory, and anti-cancer (Xu et al., 2016). Among them, the anti-tumor effect of saponins has been widely studied, and its interaction with cholesterol regulation plays an important role in its anti-tumor activity. Saponins can enhance cytotoxicity, inhibit tumor cell metastasis, reverse chemotherapeutic drug effects, and small molecule targeted drug resistance, induce immunotoxin macromolecule escape and ferroptosis by regulating cholesterol, suggesting that saponins have potential as new cancer therapeutic agents by specifically targeting cholesterol.

### 4.1 Saponin-cholesterol interaction mediated cytotoxicity

Saponins have shown good anti-cancer potential in many tumor cells by inhibiting cell growth and inducing cell apoptosis. Cytotoxicity for most saponins, seem to be ascribed to their ability to interact with membrane lipids, especially cholesterol.

Park et al. (2010) found that Rh2 induced lipid raft internalization, which caused cell membrane Akt protein inactivation and finally induced cell apoptosis. The study demonstrated that Rh2 can modulate the cell membrane cholesterol levels and influence cell viability regardless of cholesterol addition in MDA cells. Verstraeten et al. (2018) also reported that membrane cholesterol can delay the activity of ginsenoside Rh2, suggesting that saponin cytotoxicity is linked to its interaction with membrane cholesterol. Changhong et al. (2021) indicated that Ginsenoside Rb1 could inhibit the toxicity of A-β25-35-induced pheochromocytoma (PC12) cells, and could produce protective effects, including inhibiting cell growth and inducing cell apoptosis. The mechanism proposed that ginsenoside Rb1 could serve as an agonist of peroxisom proliferator-activated receptor-γ (PPARγ) and therefore decrease the content of cholesterol. Additionally, other saponins have been shown to have similar effects. Polyphyllin D (PD) (Chen et al., 2023), a steroidal saponin extracted from the rhizomes of Paris polyphylla, has been proven to induce G2/M phase arrest and inhibit the growth of liver cells by disrupting cholesterol biosynthesis. Avicins D, a triterpenoid saponin, has been demonstrated by Xu et al. (2009) to induce cell apoptosis in cell lines lacking cell death receptors. Upon avicin D treatment, Fas translocate to lipid rafts containing cholesterol, where it associates with Fas-associated death domain (FADD) and Caspase-8 to form the death-inducing signaling complex (DISC). Lorent J. et al. (2014) further investigated the role of cholesterol and saponin structure in apoptosis and membrane permeabilization in two malignant monocytic cell lines, and showed that the activity of α-hederin is primarily determined by its interaction with membrane cholesterol and subsequent pore formation.

### 4.2 Effects of saponins interaction with cholesterol on tumor metastasis

The metastasis of cancer cells is a cascade of steps with biological cell progressions including dissemination and migration (Xiao et al., 2023), tumor angiogenesis, tumor microenvironment regulation and epithelial-mesenchymal transition, *etc*. Recently, saponins have been reported to be involved in the steps that constitute the cascade reaction, which appear amenable to the treatment of cancer metastasis. This is closely related to the interaction with cholesterol (Liu et al., 2021).

Barthomeuf et al. (2004) discovered that Hederacolchiside-A1 (Hcol-A1), a triterpenoid saponin from Hedera colchica Koch, has anti-melanoma activity (Kim et al., 2023) and might affect the endothelial cell network communication system required to inhibit tumor metastasis. Hcol-A1’s activity was found to be critical with plasma membrane cholesterol sequestration. Zhu et al. (2022) discovered that Pulsatilla saponin E (PSE) effectively decreased NSCLC cell survival, migration, and invasion while promoting apoptosis. This was achieved by adjusting apoptosis-related proteins, reducing the levels of free cholesterol (FC) and total cholesterol (TC), and downregulating the expression of flotillin-1, flotillin-2, Akt, and FASN in a concentration-dependent manner. However, the inhibitory impact of PSE on A549 cell survival, migration and invasion was reversed by overexpressing flotillin-2 in lipid rafts (LR), which was closely related to membrane cholesterol levels.

### 4.3 Reverse multidrug resistance

Chemotherapeutics and small-molecule targeted drugs are effective treatments for cancer. However, cancer cells can develop resistance to these drugs. The development of multidrug resistance is a major reason why cancer chemotherapy and targeted therapy fail. Interestingly, the reversal of drug resistance has been linked to the cholesterol content in the cell membrane (Yan et al., 2020; Kopecka et al., 2020). Controlling the cholesterol levels in tumor cells has emerged as a potential strategy for combating multidrug resistance in cancer and improving therapeutic outcomes. Saponins have been shown to have varying degrees of effectiveness in reversing drug resistance to chemotherapy drugs and small-molecule targeted drugs. This effectiveness is closely related to their ability to regulate cholesterol levels. Therefore, saponins have the potential to be used in combination therapies to reverse drug resistance.

It is well known that disruption of lipid rafts, which are cholesterols-rich microdomains, could alter membrane functions, suggesting that cholesterol plays a vital role in raft function (Ye et al., 2019). Yun et al. (2013) have proven that Ginsenoside Rp1 can reverse resistance to actinomycin D by reducing MDR-1 protein levels and Src phosphorylation while modulating lipid rafts, which has a better therapeutic efficacy compared to the positive drug MβCD. The addition of cholesterol attenuated the aggregation of lipid rafts induced by Rp1 and the redistribution of MDR-1. Similarly, Kwon et al. (2008) made a significant discovery by demonstrating that Rg3, a metabolite found in ginseng, has the unique ability to selectively reduce cholesterol dependent membrane fluidity and impede the accumulation of P-gp mediated drugs in multi-drug resistance (MDR) cells, while not affecting drug-sensitive cells. However, due to the hemolytic problem of saponins, the single-agent administration mode of saponins may lead to a relatively low bioavailability *in vivo*. Additionally, Ginsenosides Rg1 and CK (Qiu et al., 2022) could lead to cholesterol efflux and decrease intracellular cholesterol content by upregulating LXRαin in TMZ-resistant GBM cells, which redistributed lipid rafts and effectively regulated the resistance of GBM cells to TMZ. Polyphyllin I (Yang et al., 2018), known for its ability to coagulate cell membrane cholesterol, was reported to modulate the MALAT1/STAT3 signaling pathway, leading to apoptosis in gefitinib-resistant NSCLC cell. However, Polyphyllin I possesses relatively high toxicity and hemolytic property, which poses limitations when applied *in vivo*. Wang et al. (2022) discovered that Polyphyllin VII demonstrates potent and selective anti-multiple myeloma both *in-vitro* and *in-vivo* by binding to the cell membrane protein-moesin, resulting in a decrease in its protein levels and subsequently inhibiting the Wnt/β-catenin pathway. Consequently, Polyphyllin VII reduces the surplus population of cells and overcomes bortezomib resistance.

### 4.4 Saponin-induced RIP endolysosomal escape

Targeting toxins through ribosome inactivating proteins (RIPs) (Smith et al., 2017) has become a topic of extensive research, but their successful clinical applications as therapeutic drugs are still limited to date. One of the major factors limiting the effectiveness of these protein-based therapies is their internalization efficiency through receptor-mediated endocytosis (RME), as well as their subsequent release efficiency from lysosomal compartments into the cytoplasm, where toxin components can catalyze the action on target ribosomes. Poor internalization of the target toxin and then recycling back to the cell surface or partial transport of the toxin to the lysosome and subsequent degradation may play a role (Weng et al., 2012; Thakur et al., 2013). In a decade, the endoplasmic escape activity of triterpenoid saponins has been studied as a potential powerful tool for improving cytoplasmic penetration of protein drugs through endocytosis, thereby greatly enhancing their pharmacological effects. More importantly, cholesterol seems to play a central actor in the enhancement of toxin cytotoxicity by triterpenoid saponins (Cao et al., 2020).

Smith et al. (2017) studied the effects of plasma membrane and cellular cholesterol on the enhancement and dissolution properties of saponins on Daudi human lymphoma cell lines. The results indicated that when lipid-deprived Daudi cells supplemented their cholesterol with low-density lipoprotein (LDL), the effect of SA on enhancing BU12 SAP IT cytotoxicity depends on cholesterol (Wensley et al., 2021). Meanwhile, they suggested that SA-induced lysosome escape may depend more on the curvature of the cell membrane and the actual cholesterol content in the bilayer, and therefore how this is also influenced by membrane phospholipid content. Wang et al. (2023) found that Momordin Ic (MIC) from Kochia scoparia (L.) Schrader significantly enhanced the cytotoxicity of recombinant MAP30, a type I ribosomal inactivation protein derived from bitter melon, in breast cancer cells, including triple cancers. The presence of cholesterol and ganglioside GM1 in the cell membrane were shown to be key factors in enhancing the cytotoxicity of MAP30, while the endosomal escape activity of MIC was less important. Raddeanin A (Kang et al., 2022), an oleanane-type triterpenoid saponin, has been shown to significantly promote endosome escape because it can recruit galactose-9, an endosome escape event reporting protein. As expected, RA effectively enhanced the anti-tumor effect of MAP30. Meanwhile, the effect of RA is related to the intracellular pH value and cell membrane cholesterol content, as well as cell type indolence.

### 4.5 Ferroptosis

As a novel form of regulated cell death, ferroptosis triggers the production of reactive oxygen species (ROS) and lipid peroxidation resulting from iron overload. Consequently, the molecules that directly or indirectly modulate these mediators are expected to regulate the process of iron-induced cellular demise. Glutathione (GSH) plays a pivotal role in the antioxidant system, safeguarding against lipid peroxidation, while SLC7A11-mediated cystine uptake enhances GSH synthesis. Therefore, alterations in GSH levels and solute carrier family proteins (SLC7A11 and SLC40A1) serve as commonly employed indicators for assessing iron mediated death. Recent evidence suggests (Liu et al., 2021) that lymphoma cells reliant on lipoprotein-mediated cholesterol uptake also undergo iron mediated death—a mechanism of cell demise dependent on oxygen and iron—triggered by the accumulation of oxidized lipids within the cellular membrane unless glutathione peroxidase 4 (GPX4), an enzyme responsible for reducing these toxic lipids (Forcina and Dixon, 2019), intervenes.

Nowadays, with the increasing research in the mechanism of ferroptosis, more studies have found that saponins can induce ferroptosis through their unique regulation effect on cholesterol. Zhou et al. (2023) found that Tim-AIII, a steroid saponin, is able to decrease cholesterol levels in cancer cells. Further evidence has also shown that it is able to form a complex with HSP90 protein to target and degrade the expression of GPX4, which ultimately leads to induced ferroptosis in lung cancer cells. And Tim-AIII possessed relatively good safety when applied *in vivo*. Lu et al. (2014) found that Ruswcogenin could positively influence fatty liver changes in type 2 diabetes and in the insulin resistant state. Hence, based on its cholesterol-lowering effect, Song et al. (2020) further proved that Ruscogenin induced ferroptosis by regulating the levels of transferrin and ferroportin. Meanwhile, saponins that have coagulative properties and regulate cell membrane cholesterol have also been reported to be related to the mechanism of ferroptosis. For instance, Wu et al. (2022) showed that α-hederin could lead to ferroptosis and membrane permeabilization in non-small cell lung cancer at safe, low toxic doses by disrupting the GSS/GSH/GPX2 axis-mediated GSH redox system. Xie and Chen (2021) found that dioscin has certain effects on the expression of transferrin and ferroproteins, which could regulate iron level in cells, and thus induce ferroptosis.

## 5 The limitations of the saponins hemolytic toxicity on its anti-tumor activity

In summary, although saponins can exert better anti-tumor activity by regulating cholesterol levels and binding to cell membrane cholesterol, the hemolytic toxicity of most saponins cannot be ignored. Due to the interaction of saponins with cholesterol on the tumor cell membrane, they can likewise react with cholesterol on the red blood cell membrane, forming pores that destabilize the cell membrane, thereby leading to the rupture of red blood cells and the efflux of hemoglobin (Vo et al., 2017). Nevertheless, some saponins exhibit only weak or no hemolytic effects, which depends on both the aglycones and the number and sequence of sugar side chains in saponins (Chwalek et al., 2006). This review will also enumerate some saponins with favorable anti-tumor activities and the intensities of their hemolytic effects in Table 1. Meanwhile, multiple technological approaches are employed to modify the structure of saponins, thereby reducing their hemolytic toxicity. Moreover,it has also been reported (Zheng et al., 2019) that the hemolytic toxicity of saponins can be designed and predicted through computational model, which will also exert a favorable guiding effect on the future clinical application of saponins. The research on the drug delivery systems of various novel preparations can also deliver saponins to the tumor site more safely, as shown in Table 2.

**TABLE 1:** The hemolytic toxicity of saponins frequently utilized in clinical practice.

Saponins	Hemolytic activity HD_50_ (μg/mL)	References
Polyphyllin VII	0.93	Liu et al. (2013)
Polyphyllin II	1.11	
Polyphyllin I	3.87	
Diosgenin	>100	
gracillin	4.70	
Polyphyllin H	7.12	
Polyphyllin VI	18.24	
Protodioscin	>100	
Ginsenoside Rh2	<60	Alghareeb et al. (2024)
Ginsenoside Rg3	<30	Cong et al. (2020)
Pulsatilla Saponin D	5.75	Chen et al. (2018)
Pulsatilla Saponin A	2.89	Tong et al. (2017)
*Bupleurum chinense* saponins	>500	Sun (2006)

**TABLE 2:** Saponins that exert anti-tumor effects by regulating cholesterol levels.

Saponins	Cancer types	Cholesterol target	Antitumor mechanisms	*in vivo* experiments	References
Ginsenoside Rh2	A431, A549, THP-1, U937 cells	lipid raft	Akt, Bad, caspase-9, caspase-3	No	Thakur et al. (2013) Tuzmen et al. (2019)
Ginsenoside Rb1	PC12 cell	PPARγ	Aβ25-35-induced	No	Verstraeten et al. (2018)
Polyphyllin D	HepG2 cell	Membrane cholesterol	apoptosis	No	Wang and Tontonoz (2018)
Avicins D	Jurkat cell	lipid Rafts	Fas, Caspase-8	No	Wang et al. (2022)
α-hederin	U937, THP-1 cells	membrane cholesterol	apoptosis	Yes	Sucha et al. (2013)
			ferroptosis		Zhu et al. (2022)
hederacolchiside-A1	HUVECs	plasma membrane cholesterol	VEGFR2, HaRas/MEK/ERK	No	Wei et al. (2016)
Pulsatilla saponin E	A549 cell	flotilin-1, flotilin-2	Akt/FASN	No	Weng et al. (2020a)
Ginsenoside Rp1	OVCAR-8, DXR cells	CAV-1, GM-1	MDR-1, Scr	No	Xiao et al. (2023)
Ginsenoside Rg3	KB V20C cells	membrane fluidity	P-gp	Yes	Xie et al. (2024)
Polyphyllin I	GBM cell	membrane cholesterol	MALAT1/STAT3	Yes	Xie and Chen (2021)
Polyphyllin VII	ARP1, ANBL6, ANBL6-BTZ cells	membrane cholesterol	Wnt/β-catenin	No	Xu et al. (2020)
Ginsenosides Rg1 and CK	U251-WT, U251-R cells	LXRαin, Lipid Raft	MDR-1, MGMT	No	Nobili et al. (2020)
Saponinum album	Daudi human lymphoma cell	membrane cholesterol,LDL	BU12-SAP endolysosomal escape	No	Yang et al. (2018)
Momordin Ic	Hela cell	cholesterol, ganglioside GM1	MAP30 endolysosomal escape	No	Ye et al. (2019)
Raddeanin A	SMMC-7721, MCF-7, A549, L02 cells	Galectin-9	MAP30 endolysosomal escape	No	Yun et al. (2013)
Tim-AIII	H1299, A549, SPC-A1 cells	SREBP2	GPX4, HSP90	Yes	Zhang et al. (2021)
Ruscogenin	BxPC-3, SW 1990, PANC-1, AsPC-1 cells	cholesterol	ROS, ferrous irons	No	Zhou et al. (2023)
dioscin	A375, G361, WM115 cells	HMGCR	ferrostatin-1, GPNA, compound 968 [107]	Yes	Vo et al. (2017)

## 6 Conclusions and perspectives

The anti-tumor activities of saponins associated with cholesterol regulation have emerged as a promising area of research in recent years. The unique pharmacological properties of saponins, including their ability to regulate cholesterol homeostasis, have opened new pathways for cancer therapy. Cholesterol regulation by saponins is a complex process that involves multiple mechanisms, including modulation of cholesterol synthesis, metabolism, uptake, as well as forming soluble or insoluble complexes with cholesterol. The anti-tumor activities of saponins are believed to be, in part, a result of their ability to disrupt cholesterol homeostasis in tumor cells, triggering apoptosis, ferroptosis, reverse multidrug resistance and metastasis, as shown in Table 2.

Several preclinical studies have demonstrated the potential of saponins as anti-tumor agents, but further clinical evaluation is required to assess their safety and efficacy in cancer patients. One of the key challenges in this regard is the limited bioavailability and pharmacokinetic profiles of saponins, necessitating the development of delivery systems that can improve their therapeutic index. Additionally, issues related to toxicity and side effects also need to be addressed, as they could potentially limit the clinical utility of saponins. Despite these challenges, the field of saponin-based anti-tumor therapy has enormous potential. Future research should focus on delineating the precise mechanisms underlying the anti-tumor activities of saponins, particularly their interactions with cholesterol regulation pathways. Furthermore, research should explore the potential of combining saponins with other cancer treatment modalities, such as chemotherapy, radiotherapy, and immunotherapy. Such combinations could potentially enhance the anti-tumor activity of saponins while reducing their toxicities. Additionally, innovative drug delivery systems, such as nanomedicine platforms, may provide effective solutions for improving the bioavailability and targeted delivery of saponins to tumor tissues.

In conclusion, the anti-tumor activities of saponins associated with cholesterol regulation represent a rapidly developing field with significant therapeutic potential. Ongoing and future research efforts will likely yield important insights into the mechanisms underlying these activities, paving the way for the development of novel treatment strategies that could significantly improve outcomes in patients with cancer.
